# Characterization of the complete chloroplast genome of *Michelia maudiae* (Magnoliaceae)

**DOI:** 10.1080/23802359.2019.1623110

**Published:** 2019-07-10

**Authors:** Junqing Wang, Yanyan Li, Qiong Wang, Weiwei Fan

**Affiliations:** Pingdingshan University, Pingdingshan, China

**Keywords:** Chloroplast genome, *Michelia maudiae*, phylogenetic analysis

## Abstract

*Michelia maudiae* is an evergreen ornamental shrub with strong aromatic flowers with great important for horticulture. In this study, the complete chloroplast genome of *M. maudiae* was assembled based on the Illumina sequences. The genome was 160,154 bp in length presenting a typical quadripartite structure and contains an inverted repeat region (IR, 26,575 bp), a large single copy region (LSC, 88,200 bp) and a small single copy region (SSC, 18,804 bp). The overall GC content was 39.24%. A total of 130 genes were identified, including 83 protein-coding genes, 38 transfer RNA genes, and eight rRNA genes. Twelve gene species contained one or two introns. The maximum-likelihood phylogenetic analysis revealed that *M. maudiae* was closely related with *Manglietia insignis*.

*Michelia maudiae* is an evergreen ornamental shrub with strong aromatic flowers belonging to the magnolia family, and therefore, has always been cultivated in China for wood production. The Magnoliaceae is a family of lowering plant within the order Magnoliales (Xia et al. 2008) and is considered as one of the most primitive groups of angiosperms (Li and Guo 2014). A good understanding of them would have important implications for revealing the origin of angiosperms and the systematics and evolution of the family Magnoliaceae (Wang et al. 2010; Xia et al. 2008). Chloroplast has been a valuable tool to be used for phylogenetic studies due to its gene conservation and the lack of recombination(Lin et al. 2012; Ravi et al. 2008). Here, we assembled the cp genome of *M. maudiae* by using sequences obtained with the Illumina HiSeq platform. The annotated cpDNA has been deposited into GenBank with the accession number MK631950.

Total Genomic DNA was extracted from the fresh, young leaves of three *M. maudiae* plants found in Longzhong Botanical Garden (32°10′N, 112°10′E), Hubei, China. The specimen of *M. maudiae* was stored in the Huazhong Agricultural University. DNA was used to construct a library for sequencing with Illumina Hiseq 2500 platform (Illumina, San Diego, CA, USA). Additionally, MITObim v 1.8 (https://github.com/chrishah/MITObim) was used to assemble the complete circular cp genome sequence (Hahn et al. 2013). The cp genome was annotated and manually adjusted with CpGAVAS (Liu et al. 2012). The circular plastid genome map was completed with the help of the online program OrganellarGenome DRAW (OGDRAW) (Lohse et al. 2013), and the annotated sequence was submitted to NCBI.

The complete cp genome sequence of *M. maudiae* was 160,154 bp in length presenting a typical quadripartite structure and contains an inverted repeat region (IR, 26,575 bp), a large single copy region (LSC, 88,200 bp), and a small single copy region (SSC, 18,804 bp). The overall GC content was 39.24%. In total, 130 genes were annotated, including 83 (63.85%) protein-coding genes (PCGs), 38(29.23%) tRNA genes, eight (6.15%) rRNA genes, and one genes (0.77%) were inferred to be pseudogenes. Ten protein-coding genes (*rps12*, *atpF*, *trnL-UAA*, *trnV-UAC*, *rpl2*, *ndhB*, *trnI-GAU*, *ycf68*, *trnA-UGC*, and *ndhA*) contained one intron while *ycf3* and *clpP* each contained two introns.

To ascertain their phylogenetic placements within the family Magnoliaceae, the phylogenetic relationships was performed using the complete cp genomes of *M. maudiae* with those of obtained from 30 other species of Magnoliaceae reported in Genbank of NCBI database based on maximum-likelihood (ML) analysis using MEGA 7.0 (Kumar et al. 2016) (https://www.megasoftware.net). The result revealed that *M. maudiae* was closely related with *Manglietia insignis*, and the three taxa of the genus *Michelia* failed to form a monophyletic clade, forming a clade included in Magnolia (Figure 1). This phylogenetic result demonstrated that the magnolia species might be a polyphyletic group and was consistent with the analysis by Kim et al. (2001). The cp genome of *M. maudiae* provided valuable genomic information, taxonomy, and phylogeny programs of Magnoliaceae studies.

**Figure 1.**
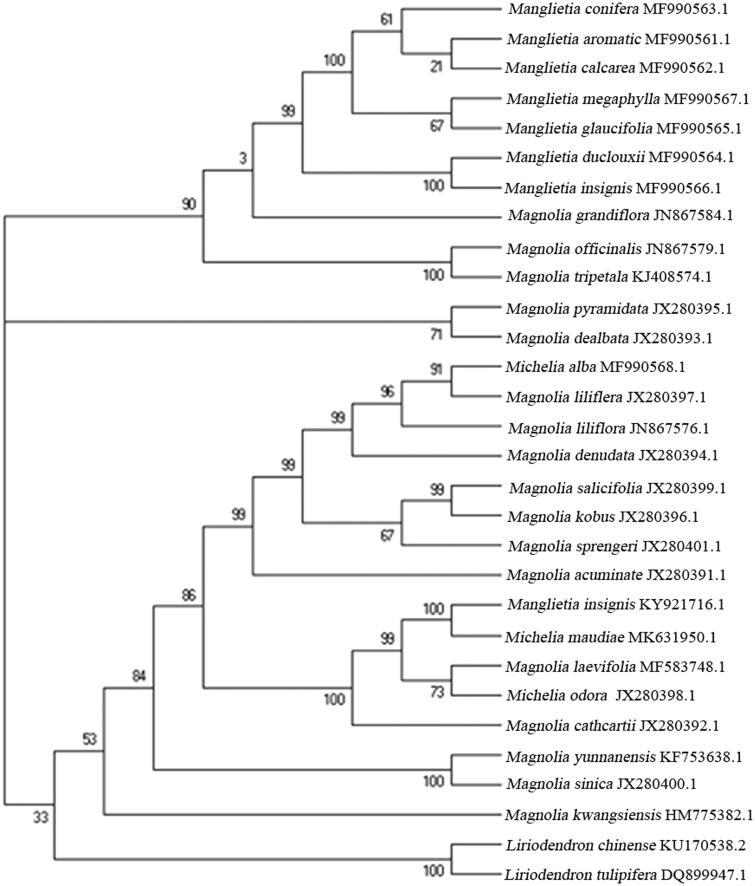
Maximum likelihood phylogenetic tree based on 29 selected Magnoliaceae chloroplast genome sequences.
